# 2653. Duration of Respiratory Virus Detection by Multipathogen PCR Testing after Acute Respiratory Illness

**DOI:** 10.1093/ofid/ofad500.2264

**Published:** 2023-11-27

**Authors:** Jennifer E Schuster, Brian R Lee, Rangaraj Selvarangan, Olivia Almendares, Sadia Sleweon, Hannah L Kirking, Jennifer Goldman

**Affiliations:** Children’s Mercy Kansas City, Kansas City, Missouri; Children's Mercy Kansas City, Kansas City, Missouri; Children’s Mercy Kansas City, Kansas City, Missouri; Centers for Disease Control and Prevention, Atlanta, Georgia; Center for Disease Control and Prevention, Atlanta, Georgia; Division of Viral Diseases, National Center for Immunization and Respiratory Diseases, CDC, Atlanta, Georgia; Children's Mercy Hospital, Kansas City, Missouri

## Abstract

**Background:**

Persons with respiratory viral infections (RVI) may shed detectable virus after symptom recovery. Duration of detection by molecular methods is not well described for non-SARS-CoV-2 respiratory viruses.

**Methods:**

School KIDS is a prospective respiratory viral surveillance program in a Missouri school district. Participating students and staff with RVI symptoms can receive respiratory viral testing using self-collected nasal swabs. Symptomatic participants with detectable virus(es) on primary swabs were scheduled to receive 2 convalescent swabs weekly after initial test during weekly school surveillance testing. All convalescent tests performed within 21 days of the primary specimen were included. Only participants providing ≥ 1 convalescent swab were included. Specimens were tested using Hologic® Panther Fusion® or QIAstat-Dx PCR assays that included adenovirus (AdV); seasonal coronaviruses (sCoV) 229E, HKU1, NL63, OC43; human metapneumovirus, influenza A and B (Flu), parainfluenza viruses 1-4, respiratory syncytial virus, rhinovirus/ enterovirus (RV/EV), and SARS-CoV-2. We used survival analysis to quantify median number of days until individuals tested negative for each virus.

**Results:**

From November 3, 2022 – April 14, 2023, 344 symptomatic participants (141 preK/elem, 49 middle, 22 high, 132 staff) submitted 586 primary specimens; 292 (50%) were positive for a virus. For positive primary specimens, median [IQR] time of symptom onset to specimen collection was 2 [1,3] days. A total of 320 viruses were detected; most common were RV/EV (37%) and sCoVs (25%) (Table). An additional 370 convalescent specimens were collected (87 specimens on days 1-7 following primary positive; 166 specimens on days 8-14; and 117 specimens on days 15-21). Median number of days [95% confidence interval] when repeat convalescent tests became negative varied by virus (AdV 15 [11,undefined] days, Flu A 14 [9,19] days, RV/EV 12 [11,14] days, SARS-CoV-2 14 [14,17] days, sCoV 10 [9,12] days) (Figure).

**Table.**
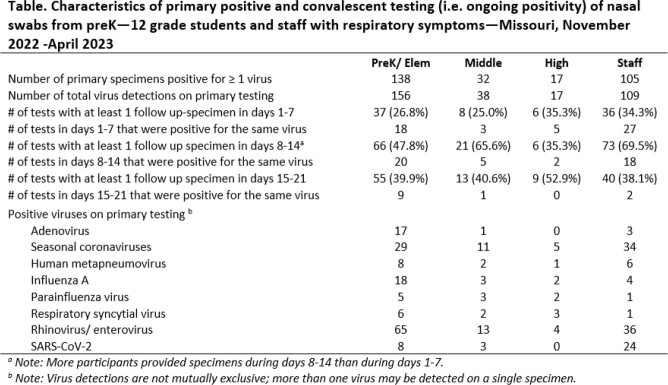
Characteristics of primary positive and convalescent testing (i.e. ongoing positivity) of nasal swabs from preK—12 grade students and staff with respiratory symptoms—Missouri, November 2022 -April 2023

**Figure.**
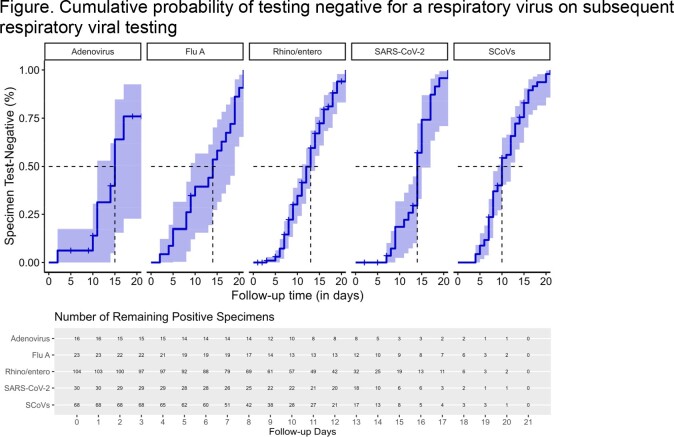
Cumulative probability of testing negative for a respiratory virus on subsequent respiratory viral testing

**Conclusion:**

Students and staff continued to have detectable virus by nasal swabs 10-15 days after symptomatic positive specimen. Further data are needed to determine when participants are no longer infectious.

**Disclosures:**

**Rangaraj Selvarangan, BVSc, PhD, D(ABMM), FIDSA, FAAM**, Abbott: Honoraria|Altona Diagnostics: Grant/Research Support|Baebies Inc: Advisor/Consultant|BioMerieux: Advisor/Consultant|BioMerieux: Grant/Research Support|Bio-Rad: Grant/Research Support|Cepheid: Grant/Research Support|GSK: Advisor/Consultant|Hologic: Grant/Research Support|Lab Simply: Advisor/Consultant|Luminex: Grant/Research Support

